# (20*S*,2′′*S*)-20-[4′-(3′′-Hydroxy-2′′-methyl­prop­yl)-3′-methylisoxazol-5-yl]-5*β*-preg­nan-3β,16β-diol

**DOI:** 10.1107/S1600536809050478

**Published:** 2009-11-28

**Authors:** María-Guadalupe Hernández Linares, Jesús Sandoval Ramírez, Socorro Meza Reyes, Sara Montiel Smith, Sylvain Bernès

**Affiliations:** a Escuela de Ingeniería Química, Universidad del Istmo, Ciudad Universitaria s/n, 70760 Sto. Domingo Tehuantepec, Oax., Mexico; b Facultad de Ciencias Químicas, Benemérita Universidad Autónoma de Puebla, Ciudad Universitaria, San Manuel, 72000 Puebla, Pue., Mexico; c DEP Facultad de Ciencias Químicas, UANL, Guerrero y Progreso S/N, Col. Treviño, 64570 Monterrey, N.L., Mexico

## Abstract

The title steroidal compound, C_29_H_47_NO_4_, was prepared in a one-pot reaction starting from a sarsasapogenin derivative of known configuration. The isoxazole heterocycle is oriented towards the α face of the steroid nucleus and, although fully functionalized on C atoms, does not provoke steric hindrance with the adjacent *D* ring. The absolute configuration observed for chiral centers is as expected, and shows that no epimerization occurred in the precursors. In the crystal, the three OH groups serve as donors for hydrogen bonding with O and N atoms. The isoxazole N atom is involved in O—H⋯N hydrogen bonds, forming chains along [100]. These chains are further connected *via* O—H⋯O and weak C—H⋯O contacts, giving rise to a three-dimensional supra­molecular network.

## Related literature

For a general introduction to steroids functionalized with heterocycles, see: Banday *et al.* (2008 ▶); Pathak & Jindal (1998 ▶); Litvinovskaya *et al.* (1998 ▶); Beam *et al.* (2000 ▶). For the biological activity of danazol, a steroid sharing structural features with the title compound, see: Gupta *et al.* (1999 ▶). For 23-acetylsarsasapogenin, used as starting material, see: Meza-Reyes *et al*. (2005 ▶).
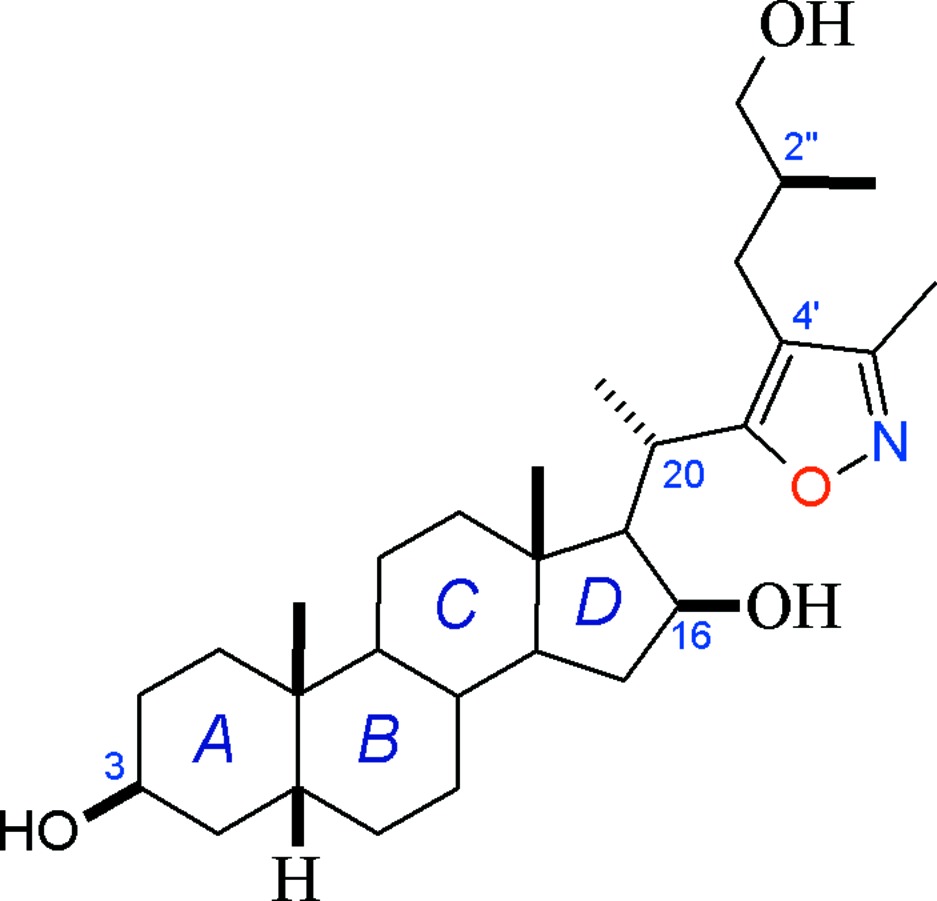


## Experimental

### 

#### Crystal data

C_29_H_47_NO_4_
*M* *_r_* = 473.68
Monoclinic, 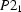

*a* = 6.5540 (8) Å
*b* = 30.131 (4) Å
*c* = 7.1971 (10) Å
β = 98.500 (13)°
*V* = 1405.6 (3) Å^3^
*Z* = 2
Mo *K*α radiation
μ = 0.07 mm^−1^
*T* = 296 K
0.6 × 0.2 × 0.2 mm

#### Data collection

Bruker P4 diffractometer
8425 measured reflections
2534 independent reflections
2003 reflections with *I* > 2σ(*I*)
*R*_int_ = 0.030

#### Refinement

*R*[*F* ^2^ > 2σ(*F* ^2^)] = 0.035
*wR*(*F* ^2^) = 0.092
*S* = 1.07
2534 reflections
322 parameters
1 restraint
H atoms treated by a mixture of independent and constrained refinement
Δρ_max_ = 0.13 e Å^−3^
Δρ_min_ = −0.12 e Å^−3^

### 

Data collection: *XSCANS* (Siemens, 1996 ▶); cell refinement: *XSCANS*; data reduction: *XSCANS*; program(s) used to solve structure: *SHELXS97* (Sheldrick, 2008 ▶); program(s) used to refine structure: *SHELXL97* (Sheldrick, 2008 ▶); molecular graphics: *SHELXTL* (Sheldrick, 2008 ▶) and *Mercury* (Macrae *et al.*, 2006 ▶); software used to prepare material for publication: *SHELXL97*.

## Supplementary Material

Crystal structure: contains datablocks I, global. DOI: 10.1107/S1600536809050478/jj2015sup1.cif

Structure factors: contains datablocks I. DOI: 10.1107/S1600536809050478/jj2015Isup2.hkl

Additional supplementary materials:  crystallographic information; 3D view; checkCIF report

## Figures and Tables

**Table 1:** Hydrogen-bond geometry (Å, °)

*D*—H⋯*A*	*D*—H	H⋯*A*	*D*⋯*A*	*D*—H⋯*A*
O32—H32⋯O33^i^	0.82 (5)	1.93 (5)	2.746 (3)	170 (5)
O33—H33⋯N24^ii^	0.85 (4)	2.03 (4)	2.828 (3)	157 (4)
O34—H34⋯O32^iii^	0.90 (6)	1.89 (6)	2.775 (4)	166 (5)
O33—H33⋯O23^ii^	0.85 (4)	2.69 (4)	3.536 (3)	171 (4)
C18—H18*C*⋯O23^ii^	0.96	2.46	3.362 (4)	157
C28—H28*C*⋯O34^iv^	0.97	2.60	3.471 (4)	150

Symmetry codes: (i) 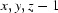; (ii) 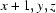; (iii) 
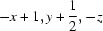
; (iv) 
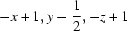
.

**Fig. 1.**
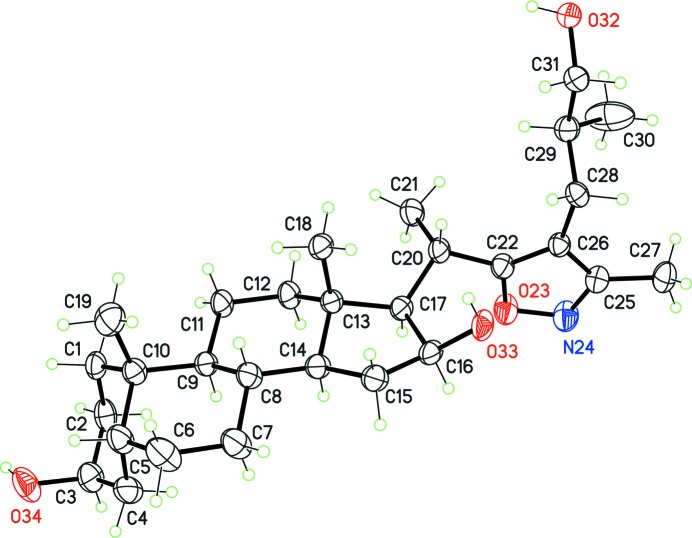
The title molecule with displacement ellipsoids for non-H atoms shown at the 30% probability level.

**Fig. 2.**
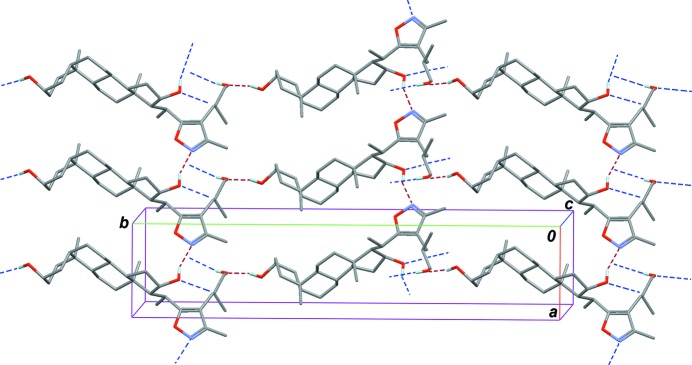
Packing diagram for (I) viewed down the c axis. Dashed lines indicate O—H···O and O—H···N intermolecular hydrogen bonds. Weak C—H···O intermolecular interactions have been omitted for clarity.

## supplementary crystallographic information

### Comment

There is a continuous interest for new synthetic routes affording steroids functionalized with heteroatoms and heterocycles, since these groups modify the biological activity of related molecules (Banday *et al.*, 2008; Pathak & Jindal, 1998). For example, the synthesis of suitable precursors for steroids analogs to brassinosteroids has been reported (Litvinovskaya *et al.*, 1998). Functionalization with an isoxazol heterocycle has been limited to date to few examples, where the heterocycle is fused with the *A* ring of the steroid. An example of a molecule belonging to this family is danazol (Gupta *et al.*, 1999), a derivative of ethisterone, which has numerous medicinal applications.

In relation with this general goal, we have developed several reagents for the direct one-step functionalization of steroids on remote positions. The title compound was synthesized readily starting from 23-acetylsarsasapogenin (Meza-Reyes *et al.*, 2005) in a one-pot reaction carried out in dry media (see *Experimental*). This new route improves known procedures, which make necessary the isolation of an oxime intermediate, prior to the heterocyclization in acid conditions (Beam *et al.*, 2000). Full details about the involved chemistry and mechanistic aspects of this unprecedented reaction will be reported elsewhere. It was however essential to X-ray characterize the product, in order to determine if any epimerization occurred during the cleavage of rings *E* and *F*.

The title molecule displays the expected *cis*-fused *A*/*B* ring system, characteristic of sarsasapogenin derivatives (Fig. 1). Rings *A*,*B* and *C* have the expected chair conformation, while the 5-membered ring *D* is twisted on C13—C14. The spiroketal *E*/*F* system was cleaved during the reaction, affording a C_21_-pregnane nucleus substituted at C20 by an isoxazol heterocycle. Positions for O and N atoms in the heterocycle were unambiguously determined from X-ray data, and are consistent with the positions for double bonds, C22=C26 and C25=N24. The isoxazol ring is oriented towards the *α* face, and its plane approximately bisects the mean plane of the *A*···*D* steroidal nucleus. This conformation avoids any hindrance with the methyl group, C21, and OH group at C16. The observed absolute configuration indicates that the *E*/*F* rings cleavage occurred without epimerization, despite use of the strongly acidic medium used for the reaction. C20 is retained as *S*, and the chiral C atom C29 in the lateral chain has the *S* configuration.

In the crystal, molecules are associated *via* O—H···N hydrogen bonds involving the isoxalic N atom as an acceptor, forming chains running along the [100] direction (Fig. 2). The hydroxyl groups at O32 and O34, form O—H···O contacts between chains. The resulting three-dimensional supramolecular network also includes weak C—H···O hydrogen bond interactions involving the O23 and O24 atoms as acceptors.

### Experimental

A mixture of 23-acetylsarsasapogenin (2 mmol), hydroxylamine hydrochloride (4 mmol) and a previously prepared P_2_O_5_/SiO_2_ reagent (1 g) were grounded thoroughly in a mortar. An immediate color change was observed. The mortar was covered with a watch glass and put inside a microwave device (2450 MHz, 1200 W). The mixture was irradiated for 3 min, allowing the reaction to complete (TLC). The mixture was then cooled to room temperature, and 10 ml of 5% aqueous HCl was added. The resulting solution was extracted with CH_2_Cl_2_ (2×10 ml) and dried over CaCl_2_. Evaporation of solvent under reduced pressure gave the pure title compound, in 81% yield. Anal. found (calc. for C_29_H_47_NO_4_): C 73.52 (75.53), H 9.98 (9.99), N 2.95 (2.95%). Single crystals were obtained by slow evaporation of an acetone solution.

### Refinement

H atoms for hydroxyl groups, H32, H33 and H34, were found in a difference map and refined freely. C-bonded H atoms were placed in idealized positions and refined using a riding approximation, with C—H bond lengths fixed to 0.96 (methyl), 0.97 (methylene) or 0.98 Å (methine). Methyl groups were allowed to rotate about their C—C bonds. Isotropic displacement parameters for H atoms were computed from displacement of carrier atoms: *U*_iso_(H) = 1.5*U*_eq_(carrier atom) for methyl and hydroxyl groups, and *U*_iso_(H) = 1.2*U*_eq_(carrier C) for other H atoms.

### Crystal data

**Table:**

C_29_H_47_NO_4_	*F*(000) = 520
*M**_r_* = 473.68	*D*_x_ = 1.119 Mg m^−^^3^
Monoclinic, *P*2_1_	Melting point: 500 K
Hall symbol: P 2yb	Mo *K*α radiation, λ = 0.71073 Å
*a* = 6.5540 (8) Å	Cell parameters from 64 reflections
*b* = 30.131 (4) Å	θ = 3.7–11.9°
*c* = 7.1971 (10) Å	µ = 0.07 mm^−^^1^
β = 98.500 (13)°	*T* = 296 K
*V* = 1405.6 (3) Å^3^	Needle, colorless
*Z* = 2	0.6 × 0.2 × 0.2 mm

### Data collection

**Table:**

Bruker P4 diffractometer	*R*_int_ = 0.030
Radiation source: fine-focus sealed tube	θ_max_ = 25.0°, θ_min_ = 2.7°
graphite	*h* = −7→7
ω scans	*k* = −35→35
8425 measured reflections	*l* = −8→8
2534 independent reflections	3 standard reflections every 97 reflections
2003 reflections with *I* > 2σ(*I*)	intensity decay: <1%

### Refinement

**Table:**

Refinement on *F*^2^	Secondary atom site location: difference Fourier map
Least-squares matrix: full	Hydrogen site location: inferred from neighbouring sites
*R*[*F*^2^ > 2σ(*F*^2^)] = 0.035	H atoms treated by a mixture of independent and constrained refinement
*wR*(*F*^2^) = 0.092	*w* = 1/[σ^2^(*F*_o_^2^) + (0.041*P*)^2^ + 0.1586*P*] where *P* = (*F*_o_^2^ + 2*F*_c_^2^)/3
*S* = 1.07	(Δ/σ)_max_ < 0.001
2534 reflections	Δρ_max_ = 0.13 e Å^−^^3^
322 parameters	Δρ_min_ = −0.12 e Å^−^^3^
1 restraint	Extinction correction: *SHELXL97* (Sheldrick, 2008), Fc^*^=kFc[1+0.001xFc^2^λ^3^/sin(2θ)]^-1/4^
0 constraints	Extinction coefficient: 0.026 (2)
Primary atom site location: structure-invariant direct methods	

### Special details

**Table:**

Experimental. Colourless crystals, m.p. 227-228°C (acetone); [α]D -106.4° (c 1.0, EtOH); IR ν max (cm-1): 3386, 3355, 3321, 2933, 2821, 1629. 1H-NMR δ: 4.12 (1H, s, H-3), 3.97 (1H, m, H-16), 3.51 and 3.47 (2H, ABX system, J3'',2'' = 6 Hz, Jgem = 11 Hz, H-3''), 3.30 (1H, dc, J20,17 = 9 Hz and J20,21 = 7 Hz, H-20), 2.47 and 2.16 (2H, dd, J1 = J2 = 8 Hz, H-1''), 2.22 (3H, s, CH3-3'), 1.28 (3H, d, J21,20 = 7 Hz, CH3-21), 0.98 (3H, s, CH3-19), 0.95 (3H, d, J = 6 Hz, CH3-2''), 0.93 (3H, s, CH3-18). 13C-NMR, δ: 10.7 (CH3-3'), 13.2 (C-18), 17.0 (C-21), 19.4 (CH3-2''), 20.9 (C-11), 24.0 (C-19), 26.1 (C-1''), 26.2 (C-7), 26.6 (C-1), 27.8 (C-12), 29.0 (C-20), 29.9 (C-2), 33.5 (C-15), 35.2 (C-6), 35.2 (C-8), 36.1 (C-9), 36.4 (C-13), 36.5 (C-5), 39.8 (C-2''), 40.4 (C-4), 42.6 (C-10), 53.9 (C-14), 58.8 (C-17), 67.0 (C-3), 67.5 (C-3''), 72.5 (C-16), 109.9 (C-5'), 159.7 (C-3', C=N), 173.0 (C-4').

### Fractional atomic coordinates and isotropic or equivalent isotropic displacement parameters (Å^2^)

**Table:**

	*x*	*y*	*z*	*U*_iso_*/*U*_eq_	
C1	0.6068 (5)	0.66437 (9)	0.3655 (4)	0.0550 (8)	
H1B	0.7108	0.6874	0.3689	0.066*	
H1C	0.5438	0.6608	0.2357	0.066*	
C2	0.4435 (5)	0.67967 (10)	0.4788 (5)	0.0647 (9)	
H2A	0.3322	0.6581	0.4669	0.078*	
H2B	0.3868	0.7077	0.4293	0.078*	
C3	0.5310 (6)	0.68522 (11)	0.6842 (5)	0.0726 (9)	
H3A	0.4185	0.6922	0.7551	0.087*	
C4	0.6348 (6)	0.64271 (11)	0.7600 (5)	0.0747 (10)	
H4A	0.5305	0.6199	0.7616	0.090*	
H4B	0.6991	0.6476	0.8887	0.090*	
C5	0.7976 (5)	0.62606 (10)	0.6460 (4)	0.0622 (8)	
H5A	0.9072	0.6484	0.6565	0.075*	
C6	0.8957 (7)	0.58263 (12)	0.7296 (5)	0.0806 (11)	
H6B	1.0192	0.5765	0.6745	0.097*	
H6C	0.9364	0.5864	0.8638	0.097*	
C7	0.7507 (7)	0.54323 (11)	0.6954 (5)	0.0737 (10)	
H7A	0.6348	0.5477	0.7629	0.088*	
H7B	0.8227	0.5166	0.7443	0.088*	
C8	0.6706 (5)	0.53673 (9)	0.4874 (4)	0.0524 (7)	
H8A	0.7884	0.5303	0.4221	0.063*	
C9	0.5657 (5)	0.57996 (9)	0.4041 (4)	0.0467 (6)	
H9D	0.4531	0.5861	0.4760	0.056*	
C10	0.7136 (4)	0.62065 (9)	0.4332 (4)	0.0504 (7)	
C11	0.4650 (5)	0.57340 (9)	0.1993 (4)	0.0531 (7)	
H11B	0.3837	0.5995	0.1593	0.064*	
H11C	0.5729	0.5711	0.1210	0.064*	
C12	0.3259 (5)	0.53243 (9)	0.1663 (4)	0.0534 (7)	
H12A	0.2800	0.5290	0.0327	0.064*	
H12B	0.2049	0.5368	0.2272	0.064*	
C13	0.4376 (4)	0.48992 (8)	0.2428 (4)	0.0448 (6)	
C14	0.5181 (5)	0.49856 (9)	0.4517 (4)	0.0519 (7)	
H14C	0.3978	0.5071	0.5098	0.062*	
C15	0.5816 (6)	0.45203 (10)	0.5279 (4)	0.0640 (9)	
H15C	0.7132	0.4433	0.4931	0.077*	
H15D	0.5907	0.4510	0.6635	0.077*	
C16	0.4078 (5)	0.42238 (9)	0.4337 (4)	0.0531 (7)	
H16B	0.3074	0.4196	0.5213	0.064*	
C17	0.2987 (4)	0.44850 (9)	0.2579 (4)	0.0476 (7)	
H17C	0.1690	0.4597	0.2923	0.057*	
C18	0.6125 (5)	0.47874 (10)	0.1303 (4)	0.0528 (7)	
H18A	0.7116	0.5024	0.1432	0.079*	
H18B	0.5567	0.4751	0.0001	0.079*	
H18C	0.6784	0.4517	0.1772	0.079*	
C19	0.8950 (5)	0.61529 (13)	0.3227 (6)	0.0778 (10)	
H19B	0.8434	0.6123	0.1913	0.117*	
H19C	0.9725	0.5893	0.3655	0.117*	
H19D	0.9827	0.6409	0.3420	0.117*	
C20	0.2387 (5)	0.41917 (9)	0.0833 (4)	0.0504 (7)	
H20B	0.3654	0.4067	0.0478	0.061*	
C21	0.1250 (5)	0.44472 (10)	−0.0881 (5)	0.0631 (8)	
H21C	0.0756	0.4241	−0.1861	0.095*	
H21D	0.2184	0.4653	−0.1331	0.095*	
H21E	0.0105	0.4606	−0.0515	0.095*	
C22	0.1037 (4)	0.38157 (9)	0.1246 (4)	0.0499 (7)	
O23	−0.0587 (3)	0.39290 (7)	0.2120 (4)	0.0703 (7)	
N24	−0.1752 (4)	0.35417 (10)	0.2322 (4)	0.0703 (8)	
C25	−0.0791 (5)	0.32195 (10)	0.1605 (4)	0.0538 (7)	
C26	0.1011 (4)	0.33757 (9)	0.0881 (4)	0.0445 (6)	
C27	−0.1567 (5)	0.27556 (11)	0.1658 (5)	0.0661 (9)	
H27D	−0.2718	0.2748	0.2340	0.099*	
H27E	−0.0487	0.2568	0.2268	0.099*	
H27F	−0.1994	0.2652	0.0399	0.099*	
C28	0.2554 (4)	0.31208 (9)	−0.0033 (4)	0.0474 (6)	
H28C	0.2499	0.2812	0.0334	0.057*	
H28D	0.3922	0.3231	0.0446	0.057*	
C29	0.2250 (5)	0.31457 (10)	−0.2173 (4)	0.0554 (7)	
H29C	0.2155	0.3459	−0.2545	0.067*	
C30	0.0285 (6)	0.2913 (2)	−0.3036 (6)	0.123 (2)	
H30B	0.0167	0.2925	−0.4381	0.185*	
H30C	−0.0882	0.3059	−0.2641	0.185*	
H30D	0.0325	0.2609	−0.2633	0.185*	
C31	0.4103 (5)	0.29436 (10)	−0.2882 (4)	0.0624 (8)	
H31C	0.5325	0.3107	−0.2351	0.075*	
H31D	0.4266	0.2641	−0.2429	0.075*	
O32	0.3985 (5)	0.29406 (8)	−0.4864 (3)	0.0788 (8)	
H32	0.411 (8)	0.3187 (17)	−0.533 (7)	0.118*	
O33	0.4750 (3)	0.37840 (6)	0.3970 (3)	0.0570 (6)	
H33	0.584 (6)	0.3790 (14)	0.345 (5)	0.086*	
O34	0.6814 (5)	0.71965 (8)	0.7127 (4)	0.0898 (9)	
H34	0.633 (9)	0.743 (2)	0.640 (8)	0.135*	

### Atomic displacement parameters (Å^2^)

**Table:**

	*U*^11^	*U*^22^	*U*^33^	*U*^12^	*U*^13^	*U*^23^
C1	0.0655 (19)	0.0375 (15)	0.0597 (18)	−0.0086 (14)	0.0013 (15)	0.0075 (13)
C2	0.0618 (19)	0.0362 (16)	0.093 (3)	0.0006 (14)	0.0010 (18)	0.0017 (16)
C3	0.095 (3)	0.0424 (17)	0.083 (2)	0.0033 (18)	0.023 (2)	−0.0076 (16)
C4	0.121 (3)	0.0451 (18)	0.058 (2)	0.007 (2)	0.011 (2)	−0.0015 (15)
C5	0.079 (2)	0.0448 (17)	0.0573 (19)	0.0017 (15)	−0.0073 (16)	0.0000 (14)
C6	0.103 (3)	0.059 (2)	0.069 (2)	0.020 (2)	−0.025 (2)	−0.0043 (16)
C7	0.113 (3)	0.0437 (17)	0.0565 (19)	0.0123 (18)	−0.0130 (19)	0.0027 (15)
C8	0.0696 (18)	0.0367 (14)	0.0490 (15)	0.0109 (14)	0.0029 (14)	0.0027 (12)
C9	0.0567 (16)	0.0361 (14)	0.0479 (15)	0.0046 (12)	0.0099 (13)	0.0014 (11)
C10	0.0530 (16)	0.0428 (16)	0.0547 (17)	0.0034 (13)	0.0049 (13)	0.0003 (13)
C11	0.0653 (18)	0.0342 (14)	0.0564 (17)	0.0034 (13)	−0.0019 (14)	0.0071 (12)
C12	0.0599 (17)	0.0362 (14)	0.0618 (18)	0.0071 (14)	0.0009 (14)	0.0048 (13)
C13	0.0552 (16)	0.0327 (14)	0.0479 (15)	0.0084 (12)	0.0124 (13)	0.0024 (11)
C14	0.0733 (19)	0.0364 (14)	0.0471 (16)	0.0132 (13)	0.0124 (14)	0.0045 (12)
C15	0.103 (3)	0.0400 (16)	0.0488 (16)	0.0108 (17)	0.0088 (17)	0.0070 (13)
C16	0.075 (2)	0.0361 (15)	0.0540 (16)	0.0108 (14)	0.0284 (15)	0.0070 (13)
C17	0.0568 (16)	0.0341 (14)	0.0555 (16)	0.0111 (12)	0.0205 (13)	0.0027 (12)
C18	0.0611 (17)	0.0425 (15)	0.0582 (17)	0.0037 (14)	0.0198 (14)	0.0022 (13)
C19	0.064 (2)	0.083 (3)	0.090 (3)	−0.009 (2)	0.0209 (19)	−0.003 (2)
C20	0.0600 (17)	0.0341 (14)	0.0601 (17)	0.0051 (13)	0.0185 (14)	0.0018 (13)
C21	0.079 (2)	0.0459 (17)	0.0633 (19)	0.0029 (16)	0.0076 (16)	0.0018 (14)
C22	0.0503 (16)	0.0408 (15)	0.0615 (17)	0.0074 (13)	0.0175 (13)	0.0019 (13)
O23	0.0679 (14)	0.0463 (12)	0.1057 (18)	0.0066 (11)	0.0431 (13)	−0.0007 (11)
N24	0.0619 (16)	0.0570 (16)	0.099 (2)	0.0012 (14)	0.0343 (15)	0.0019 (15)
C25	0.0506 (16)	0.0491 (17)	0.0618 (18)	−0.0001 (13)	0.0082 (13)	0.0058 (14)
C26	0.0462 (15)	0.0391 (14)	0.0484 (15)	0.0026 (12)	0.0076 (12)	0.0026 (12)
C27	0.0631 (19)	0.0566 (19)	0.077 (2)	−0.0153 (16)	0.0056 (16)	0.0095 (16)
C28	0.0504 (15)	0.0388 (14)	0.0523 (16)	0.0052 (12)	0.0055 (12)	−0.0025 (12)
C29	0.0638 (18)	0.0488 (16)	0.0545 (17)	0.0065 (14)	0.0117 (14)	0.0048 (13)
C30	0.077 (2)	0.224 (7)	0.065 (2)	−0.029 (3)	0.000 (2)	−0.032 (3)
C31	0.087 (2)	0.0444 (17)	0.0610 (19)	0.0047 (16)	0.0275 (16)	0.0000 (15)
O32	0.139 (2)	0.0432 (12)	0.0641 (14)	−0.0046 (14)	0.0467 (14)	0.0002 (11)
O33	0.0660 (13)	0.0314 (10)	0.0785 (14)	0.0066 (9)	0.0266 (11)	0.0099 (10)
O34	0.128 (2)	0.0423 (13)	0.0880 (19)	−0.0053 (14)	−0.0196 (17)	−0.0085 (12)

### Geometric parameters (Å, °)

**Table:**

C1—C2	1.511 (5)	C16—O33	1.433 (3)
C1—C10	1.537 (4)	C16—C17	1.569 (4)
C1—H1B	0.9700	C16—H16B	0.9800
C1—H1C	0.9700	C17—C20	1.539 (4)
C2—C3	1.514 (5)	C17—H17C	0.9800
C2—H2A	0.9700	C18—H18A	0.9600
C2—H2B	0.9700	C18—H18B	0.9600
C3—O34	1.425 (4)	C18—H18C	0.9600
C3—C4	1.514 (5)	C19—H19B	0.9600
C3—H3A	0.9800	C19—H19C	0.9600
C4—C5	1.524 (5)	C19—H19D	0.9600
C4—H4A	0.9700	C20—C22	1.494 (4)
C4—H4B	0.9700	C20—C21	1.549 (4)
C5—C6	1.540 (5)	C20—H20B	0.9800
C5—C10	1.558 (4)	C21—H21C	0.9600
C5—H5A	0.9800	C21—H21D	0.9600
C6—C7	1.518 (5)	C21—H21E	0.9600
C6—H6B	0.9700	C22—C26	1.351 (4)
C6—H6C	0.9700	C22—O23	1.358 (3)
C7—C8	1.524 (4)	O23—N24	1.414 (3)
C7—H7A	0.9700	N24—C25	1.304 (4)
C7—H7B	0.9700	C25—C26	1.438 (4)
C8—C14	1.521 (4)	C25—C27	1.490 (4)
C8—C9	1.551 (4)	C26—C28	1.497 (4)
C8—H8A	0.9800	C27—H27D	0.9600
C9—C11	1.536 (4)	C27—H27E	0.9600
C9—C10	1.558 (4)	C27—H27F	0.9600
C9—H9D	0.9800	C28—C29	1.525 (4)
C10—C19	1.534 (4)	C28—H28C	0.9700
C11—C12	1.532 (4)	C28—H28D	0.9700
C11—H11B	0.9700	C29—C31	1.513 (4)
C11—H11C	0.9700	C29—C30	1.517 (5)
C12—C13	1.536 (4)	C29—H29C	0.9800
C12—H12A	0.9700	C30—H30B	0.9600
C12—H12B	0.9700	C30—H30C	0.9600
C13—C18	1.535 (4)	C30—H30D	0.9600
C13—C14	1.540 (4)	C31—O32	1.417 (4)
C13—C17	1.558 (4)	C31—H31C	0.9700
C14—C15	1.540 (4)	C31—H31D	0.9700
C14—H14C	0.9800	O32—H32	0.82 (5)
C15—C16	1.525 (5)	O33—H33	0.85 (4)
C15—H15C	0.9700	O34—H34	0.90 (6)
C15—H15D	0.9700		
			
C2—C1—C10	114.6 (3)	C16—C15—H15C	111.1
C2—C1—H1B	108.6	C14—C15—H15C	111.1
C10—C1—H1B	108.6	C16—C15—H15D	111.1
C2—C1—H1C	108.6	C14—C15—H15D	111.1
C10—C1—H1C	108.6	H15C—C15—H15D	109.1
H1B—C1—H1C	107.6	O33—C16—C15	113.2 (3)
C1—C2—C3	111.4 (3)	O33—C16—C17	115.5 (2)
C1—C2—H2A	109.4	C15—C16—C17	106.6 (2)
C3—C2—H2A	109.4	O33—C16—H16B	107.0
C1—C2—H2B	109.4	C15—C16—H16B	107.0
C3—C2—H2B	109.4	C17—C16—H16B	107.0
H2A—C2—H2B	108.0	C20—C17—C13	119.0 (2)
O34—C3—C2	112.0 (3)	C20—C17—C16	113.6 (2)
O34—C3—C4	107.5 (3)	C13—C17—C16	104.9 (2)
C2—C3—C4	110.1 (3)	C20—C17—H17C	106.2
O34—C3—H3A	109.1	C13—C17—H17C	106.2
C2—C3—H3A	109.1	C16—C17—H17C	106.2
C4—C3—H3A	109.1	C13—C18—H18A	109.5
C3—C4—C5	113.4 (3)	C13—C18—H18B	109.5
C3—C4—H4A	108.9	H18A—C18—H18B	109.5
C5—C4—H4A	108.9	C13—C18—H18C	109.5
C3—C4—H4B	108.9	H18A—C18—H18C	109.5
C5—C4—H4B	108.9	H18B—C18—H18C	109.5
H4A—C4—H4B	107.7	C10—C19—H19B	109.5
C4—C5—C6	110.8 (3)	C10—C19—H19C	109.5
C4—C5—C10	112.9 (3)	H19B—C19—H19C	109.5
C6—C5—C10	111.2 (3)	C10—C19—H19D	109.5
C4—C5—H5A	107.2	H19B—C19—H19D	109.5
C6—C5—H5A	107.2	H19C—C19—H19D	109.5
C10—C5—H5A	107.2	C22—C20—C17	111.1 (2)
C7—C6—C5	112.7 (3)	C22—C20—C21	107.8 (2)
C7—C6—H6B	109.1	C17—C20—C21	113.6 (2)
C5—C6—H6B	109.1	C22—C20—H20B	108.1
C7—C6—H6C	109.1	C17—C20—H20B	108.1
C5—C6—H6C	109.1	C21—C20—H20B	108.1
H6B—C6—H6C	107.8	C20—C21—H21C	109.5
C6—C7—C8	112.1 (3)	C20—C21—H21D	109.5
C6—C7—H7A	109.2	H21C—C21—H21D	109.5
C8—C7—H7A	109.2	C20—C21—H21E	109.5
C6—C7—H7B	109.2	H21C—C21—H21E	109.5
C8—C7—H7B	109.2	H21D—C21—H21E	109.5
H7A—C7—H7B	107.9	C26—C22—O23	110.4 (3)
C14—C8—C7	112.7 (3)	C26—C22—C20	134.1 (3)
C14—C8—C9	109.1 (2)	O23—C22—C20	115.4 (2)
C7—C8—C9	109.9 (2)	C22—O23—N24	108.2 (2)
C14—C8—H8A	108.4	C25—N24—O23	105.9 (2)
C7—C8—H8A	108.4	N24—C25—C26	111.7 (3)
C9—C8—H8A	108.4	N24—C25—C27	120.1 (3)
C11—C9—C8	111.5 (2)	C26—C25—C27	128.1 (3)
C11—C9—C10	113.6 (2)	C22—C26—C25	103.8 (2)
C8—C9—C10	112.2 (2)	C22—C26—C28	126.8 (3)
C11—C9—H9D	106.3	C25—C26—C28	129.5 (3)
C8—C9—H9D	106.3	C25—C27—H27D	109.5
C10—C9—H9D	106.3	C25—C27—H27E	109.5
C19—C10—C1	106.1 (3)	H27D—C27—H27E	109.5
C19—C10—C5	109.4 (3)	C25—C27—H27F	109.5
C1—C10—C5	107.5 (2)	H27D—C27—H27F	109.5
C19—C10—C9	111.2 (2)	H27E—C27—H27F	109.5
C1—C10—C9	112.7 (2)	C26—C28—C29	115.0 (2)
C5—C10—C9	109.7 (2)	C26—C28—H28C	108.5
C12—C11—C9	114.4 (2)	C29—C28—H28C	108.5
C12—C11—H11B	108.7	C26—C28—H28D	108.5
C9—C11—H11B	108.7	C29—C28—H28D	108.5
C12—C11—H11C	108.7	H28C—C28—H28D	107.5
C9—C11—H11C	108.7	C31—C29—C30	110.6 (3)
H11B—C11—H11C	107.6	C31—C29—C28	109.2 (2)
C11—C12—C13	112.0 (2)	C30—C29—C28	111.5 (3)
C11—C12—H12A	109.2	C31—C29—H29C	108.5
C13—C12—H12A	109.2	C30—C29—H29C	108.5
C11—C12—H12B	109.2	C28—C29—H29C	108.5
C13—C12—H12B	109.2	C29—C30—H30B	109.5
H12A—C12—H12B	107.9	C29—C30—H30C	109.5
C18—C13—C12	110.3 (2)	H30B—C30—H30C	109.5
C18—C13—C14	112.2 (2)	C29—C30—H30D	109.5
C12—C13—C14	106.6 (2)	H30B—C30—H30D	109.5
C18—C13—C17	110.5 (2)	H30C—C30—H30D	109.5
C12—C13—C17	116.1 (2)	O32—C31—C29	114.5 (3)
C14—C13—C17	100.8 (2)	O32—C31—H31C	108.6
C8—C14—C13	114.5 (2)	C29—C31—H31C	108.6
C8—C14—C15	119.5 (3)	O32—C31—H31D	108.6
C13—C14—C15	103.3 (2)	C29—C31—H31D	108.6
C8—C14—H14C	106.2	H31C—C31—H31D	107.6
C13—C14—H14C	106.2	C31—O32—H32	114 (3)
C15—C14—H14C	106.2	C16—O33—H33	111 (3)
C16—C15—C14	103.3 (3)	C3—O34—H34	108 (4)
			
C10—C1—C2—C3	57.4 (3)	C18—C13—C14—C15	−71.8 (3)
C1—C2—C3—O34	64.8 (3)	C12—C13—C14—C15	167.4 (3)
C1—C2—C3—C4	−54.7 (4)	C17—C13—C14—C15	45.8 (3)
O34—C3—C4—C5	−68.1 (4)	C8—C14—C15—C16	−169.8 (2)
C2—C3—C4—C5	54.2 (4)	C13—C14—C15—C16	−41.3 (3)
C3—C4—C5—C6	−179.6 (3)	C14—C15—C16—O33	148.1 (2)
C3—C4—C5—C10	−54.1 (4)	C14—C15—C16—C17	20.0 (3)
C4—C5—C6—C7	72.2 (4)	C18—C13—C17—C20	−42.3 (3)
C10—C5—C6—C7	−54.2 (4)	C12—C13—C17—C20	84.3 (3)
C5—C6—C7—C8	55.6 (4)	C14—C13—C17—C20	−161.1 (2)
C6—C7—C8—C14	−177.6 (3)	C18—C13—C17—C16	86.1 (3)
C6—C7—C8—C9	−55.7 (4)	C12—C13—C17—C16	−147.3 (2)
C14—C8—C9—C11	−50.7 (3)	C14—C13—C17—C16	−32.7 (2)
C7—C8—C9—C11	−174.7 (3)	O33—C16—C17—C20	12.9 (3)
C14—C8—C9—C10	−179.4 (2)	C15—C16—C17—C20	139.6 (3)
C7—C8—C9—C10	56.6 (3)	O33—C16—C17—C13	−118.7 (2)
C2—C1—C10—C19	−170.8 (3)	C15—C16—C17—C13	8.0 (3)
C2—C1—C10—C5	−53.8 (3)	C13—C17—C20—C22	−179.3 (2)
C2—C1—C10—C9	67.2 (3)	C16—C17—C20—C22	56.4 (3)
C4—C5—C10—C19	166.0 (3)	C13—C17—C20—C21	−57.6 (3)
C6—C5—C10—C19	−68.8 (4)	C16—C17—C20—C21	178.2 (3)
C4—C5—C10—C1	51.2 (3)	C17—C20—C22—C26	−134.1 (3)
C6—C5—C10—C1	176.4 (3)	C21—C20—C22—C26	100.9 (4)
C4—C5—C10—C9	−71.8 (3)	C17—C20—C22—O23	48.8 (3)
C6—C5—C10—C9	53.5 (4)	C21—C20—C22—O23	−76.2 (3)
C11—C9—C10—C19	−62.0 (3)	C26—C22—O23—N24	−0.6 (3)
C8—C9—C10—C19	65.5 (3)	C20—C22—O23—N24	177.2 (2)
C11—C9—C10—C1	57.0 (3)	C22—O23—N24—C25	0.9 (3)
C8—C9—C10—C1	−175.5 (2)	O23—N24—C25—C26	−0.8 (3)
C11—C9—C10—C5	176.8 (2)	O23—N24—C25—C27	177.5 (3)
C8—C9—C10—C5	−55.7 (3)	O23—C22—C26—C25	0.1 (3)
C8—C9—C11—C12	49.7 (3)	C20—C22—C26—C25	−177.1 (3)
C10—C9—C11—C12	177.6 (2)	O23—C22—C26—C28	−178.8 (2)
C9—C11—C12—C13	−53.1 (3)	C20—C22—C26—C28	4.0 (5)
C11—C12—C13—C18	−66.6 (3)	N24—C25—C26—C22	0.5 (4)
C11—C12—C13—C14	55.4 (3)	C27—C25—C26—C22	−177.7 (3)
C11—C12—C13—C17	166.7 (2)	N24—C25—C26—C28	179.3 (3)
C7—C8—C14—C13	−178.4 (3)	C27—C25—C26—C28	1.2 (5)
C9—C8—C14—C13	59.3 (3)	C22—C26—C28—C29	−81.4 (4)
C7—C8—C14—C15	−55.2 (4)	C25—C26—C28—C29	100.0 (3)
C9—C8—C14—C15	−177.6 (3)	C26—C28—C29—C31	170.2 (2)
C18—C13—C14—C8	59.7 (3)	C26—C28—C29—C30	−67.2 (4)
C12—C13—C14—C8	−61.1 (3)	C30—C29—C31—O32	55.9 (4)
C17—C13—C14—C8	177.3 (2)	C28—C29—C31—O32	179.0 (3)

### Hydrogen-bond geometry (Å, °)

**Table:**

*D*—H···*A*	*D*—H	H···*A*	*D*···*A*	*D*—H···*A*
O32—H32···O33^i^	0.82 (5)	1.93 (5)	2.746 (3)	170 (5)
O33—H33···N24^ii^	0.85 (4)	2.03 (4)	2.828 (3)	157 (4)
O34—H34···O32^iii^	0.90 (6)	1.89 (6)	2.775 (4)	166 (5)
O33—H33···O23^ii^	0.85 (4)	2.69 (4)	3.536 (3)	171 (4)
C18—H18C···O23^ii^	0.96	2.46	3.362 (4)	157
C28—H28C···O34^iv^	0.97	2.60	3.471 (4)	150

Symmetry codes: (i) *x*, *y*, *z*−1; (ii) *x*+1, *y*, *z*; (iii) −*x*+1, *y*+1/2, −*z*; (iv) −*x*+1, *y*−1/2, −*z*+1.
